# Proceedings of 3rd Australian Lipid Meeting

**DOI:** 10.3390/metabo7020016

**Published:** 2017-05-01

**Authors:** Simon H. J. Brown, Thusitha W. T. Rupasinghe

**Affiliations:** 1 School of Biological Sciences, Illawarra Health and Medical Research Institute, University of Wollongong, Wollongong 2522, Australia; simonb@uow.edu.au; 2 Metabolomics Australia, School of Biosciences, The University of Melbourne, Melbourne 3010, Australia

## The Baker IDI Heart and Diabetes Institute, Melbourne, 21–22 November 2016

More than 100 lipid researchers from across Australia participated in the 3rd Australian Lipid Meeting (ALM3), held on the 21st and 22nd of November 2016 in Melbourne, Australia. The conference was organized by Associate Professor Peter Meikle, a senior research fellow at the Baker Heart and Diabetes Institute, and supported by expertise in lipid research from Professor Ute Roessner, Professor Gavin Reid, Associate Professor Clinton Bruce, Dr. Thusitha Rupasinghe, Dr. Anthony Don, Dr. Andrew Hoy, and Dr. Simon Brown.

The 1st Australian Lipid Meeting was held in 2013, and since then the Australian lipid research community has proven to be well connected despite the geographical separation between major cities. Interest in lipid research is expanding in Australia with a substantial increase in participation observed at ALM3. The meeting was highlighted with much discussion about new technologies, realizations of common ground and commiserations of common difficulties in lipid research. The meeting created new opportunities for young scientists to expand their knowledge through expert advice as well as learning big picture concepts for future research. ALM3 was specifically designed to give extensive opportunities for students and early career researchers. Two sessions were focused towards younger researchers, including one session of six lightning talks and one session of student presentations selected from the abstracts.

The meeting was coordinated to cover seven topics including integrative lipidomics; metabolic diseases; local developments; neurodegeneration; plant and microbial lipidomics; fluxomics; and lipid signaling. The meeting was highlighted by keynote presentations from three leading international lipid experts. The meeting opened with an integrative lipidomics and data analysis session on Monday 21st November 2016, commenced by a presentation from Associate Professor Daniel K Nomura from the University of California, Berkeley. Professor Nomura discussed the use of chemoproteomic and metabolomics platforms to map drivers of human disease. This was followed by Dr. Brian Drew, discussing the analysis of the genetic regulation of the lipidome and proteome in murine liver, by quantification of more than 5000 proteins with excellent reproducibility within strains and significant variance of more than 2500 proteins between the 107 strains. In addition, quantitative lipidomics analysis of 311 lipid species across 23 lipid classes in which significant variation was shown in more than 100 lipid species and presented identification of numerous novel proteins that associate with an accumulation of pathological lipids in the liver, together with many genetic and protein signatures that define hepatic lipid metabolism. Dr. Magdalene Montgomery presented the identification of the medium chain fatty acid receptor GPR84 as a new player in glucose tolerance and mitochondrial function, concluding that the MCFA receptor GPR84 plays an important role in glucose tolerance, potentially via the regulation of mitochondrial function in skeletal muscle. Dr. Timothy Couttas introduced the bio-mechanism of glucose tolerance and the process of myelin biosynthesis related to Alzheimer’s Disease.

The second session in ALM3 was focused on metabolic diseases and how lipid analysis can be used to investigate relevant mechanisms and identify biomarkers. Professor Lisa Horvath from Chris O’Brien Lifehouse presented an identified and validated novel plasma lipid signature associated with poor prognosis in castration-resistant prostate cancer. Professor Matthew Watt from Monash University presented how the protein perilipin 5 (PLIN5) regulates lipid metabolism and insulin action using cultured cells and mice with whole-body and tissue-specific ablation of PLIN5. His study revealed that unique PLIN5 mediated remodelling of lipid metabolism in skeletal muscle and altered cellular stress signalling that impacts on systemic glucose metabolism through metabolic cross-talk. Yow-Keat Tham from Baker IDI institute, Melbourne presented a characterization of lipid modification in the heart of a transgenic mouse model with differential PI3K activity, while Aikaternini Emmanoulid from Curtin University discussed how lysophosphatidylinositol (LPI) triggers signaling in cell proliferation, migration, and survival in pancreatic cancer and the importance of the release of LPI by pancreatic cancer ductal adenocarcinoma (PDAC) cells as this molecule could be used in the future as a bio-marker to allow an early diagnosis.

A highlight of the student presentation session was Matthew Summers’ presentation, who won the best student presentation for his research work on a lipidomic approach to investigate the possibility of rescuing neurofibromatosis Type 1 (NF1) genetic disorder by dietary intervention. His findings show strong evidence that lipid storage myopathy underlies muscle weakness in NF1, and that simple dietary modification may be sufficient to dramatically improve muscle function and patient quality of life.

The Lightning talks gave an opportunity for six talented early-career researchers to present their findings in lipidomic research in five-minute long talks. Dr. Sarah Abbott presented phospholipid diversity in human red blood cells (RBC), followed by an analytical development with ozonolysis for phosphatidylcholine species in RBC by Dr. Amani Batarseh. Dr. Zhiqian Liu presented how heat stress in dairy cattle could impact on the lipid composition of cow milk; then, Dr. Husna Begum showed the application of plasma lipid profile in a population of healthy Singaporeans, leading to the identification of ethnic differences in lipid levels and association with health risk factors. Dr. Sarah Hancock showed a structural elucidation of hydroxyl fatty acid and Dr. Jeniffer Saville presented an interrogation of brain gangliosides in a neurodegenerative mouse model.

The last session of the first day was focused on local developments in lipidomics. Presentations were given on untargeted lipidomics analysis using LC-MS/MS within a mass spectrometry facility by Dr. Russell Pickford; comprehensive targeted plasma lipidomics by Kevin Huynh; bovine milk lipidomics by Dr. Zhiqing Liu; shotgun lipidomics analysis using ultra-high resolution mass spectrometry by Dr. Eileen Ryan; tools and techniques for structural characterization to investigate lipid–protein interactions by Dr. Simon Brown; and analysis of cellular lipids using deuterated water and GC-MS by Dr. Greg Kowalski.

The second day of the ALM3 meeting featured two international speakers from the USA: Professor Michelle Mielke from the Mayo Clinic and Dr. Stephen Previs from Merck. The first session of the second day focused on neurodegeneration and Professor Mielke presented her thought-provoking research findings on how sphingolipids play critical roles in pathways for multiple neurodegenerative diseases. Professor Mielke discussed the translation of the work from basic science to clinical and epidemiological studies, with a focus on the relationship between sphingolipids and Alzheimer’s disease (AD), Parkinson’s disease (PD), Lewy Body Dementia (LBD), and Multiple Sclerosis (MS), and PD pathology and clinical phenotypes, then explored how plasma and sphingolipids may be best utilized as biomarkers for the development and progression of these neurodegenerative diseases. Presentations were continued by Professor Brett Garner, discussing ATP-binding cassette transporter roles in Alzheimer's disease; Professor Frederic Meunier, discussing the relationship of free fatty acids and lysophospholipids in brain and neuronal communication and memory. The neurodegeneration session was concluded by Professor John Mamo, discussing the effect of long-chain saturated fatty acid enriched diet on the brain lipid status. His findings demonstrated that cerebral abundance of some specific lipid species is strongly associated with plasma lipid homeostasis.

The combined plant and microbial lipidomics session was opened by Dr. Surinder Singh from CSIRO, presenting the development of metabolically engineered plants that produce long-chain polyunsaturated fatty acids in plant seeds. Dr. Thusitha Rupasinghe presented an omics approach to identify salinity tolerance mechanisms in barley roots by measurement of changes in the spatial distribution of lipids due to salinity and showed insight into novel mechanisms responsible for salt tolerance of barley. Stephen Klatt discussed microbial lipidomics and the development of a comprehensive lipidomics pipeline for analyzing corynebacterium glutamicum inner and outer membrane lipids and cell wall synthesis.

International speaker, Dr. Stephen Previs, opened up a very interesting session on fluxomics, presenting an application of fluxomics in drug discovery. Dr. Andrew Jenner discussed how sterol distribution in human lens changes with aging and Dr. Ahrathy Selathurai showed the role of mitochondrial phosphatidylethanolamine synthesis in the regulation of muscle and mitochondrial structure. The final session of the meeting was focused on lipid signaling. Professor Stuart Pitson opened the session, presenting an approach to target myeloma by a synergetic combination of sphingosine kinase 2 inhibition and bortezomib. Dr. Kieran Scott discussed the relationship of novel secreted phospholipase A2 inhibitors and prostate tumor and Associate Professor Anthony Don presented how selective inhibition of ceramide synthase 1 promotes fat metabolism.

During the breaks between sessions, there was ample opportunity for networking across the small yet diverse Australian lipid research community. Attendees had the chance to get information and interact with commercial companies and suppliers for chemicals and instruments. A welcome reception and poster walk were held on the first day, followed by the conference dinner held at Albert Park in Melbourne. The lipid meeting was supported by generous sponsorships, especially from Waters, Shimadzu and Thermo-Fisher as gold sponsors, Sapphire Bioscience, Sciex and Agilent as silver sponsors. Avanti polar lipids sponsored the welcome reception while the poster prize was sponsored by Metabolomics Australia. The Australian Lipid Meeting 3 would not have been successful without the delegates of those who attended, presented and participated in valuable discussions. Our sincere thanks to all sponsors and delegates as well as invited speakers who made this event memorable. We look forward to meeting with you all at the next lipid meeting which will be held in the year 2018.

